# Peptidoglycan compositional analysis of *Mycobacterium smegmatis* using high-resolution LC–MS

**DOI:** 10.1038/s41598-022-15324-1

**Published:** 2022-06-30

**Authors:** Binayak Rimal, Sibusiso Senzani, Christopher Ealand, Gyanu Lamichhane, Bavesh Kana, Sung Joon Kim

**Affiliations:** 1 Institute of Biomedical Studies, Baylor University, Waco, TX 76798 USA; 2 Division of Infectious Diseases, School of Medicine, Johns Hopkins University, Baltimore, MD 21287 USA; 3 National Health Laboratory Service, Faculty of Health Sciences, DST/NRF Centre of Excellence for Biomedical TB Research, School of Pathology, University of the Witwatersrand, Johannesburg, 2001 South Africa; 4 Department of Chemistry, Howard University, Chemistry Building, 525 College Street, Washington, DC 20059 USA

**Keywords:** Biochemistry, Microbiology, Diseases, Chemistry

## Abstract

Peptidoglycan (PG) is the exoskeleton of bacterial cells and is required for their viability, growth, and cell division. Unlike most bacteria, mycobacteria possess an atypical PG characterized by a high degree of unique linkages and chemical modifications which most likely serve as important determinants of virulence and pathogenesis in mycobacterial diseases. Despite this important role, the chemical composition and molecular architecture of mycobacterial PG have yet to be fully determined. Here we determined the chemical composition of PG from *Mycobacterium smegmatis* using high-resolution liquid chromatography-mass spectrometry. Purified cell walls from the stationary phase were digested with mutanolysin and compositional analysis was performed on 130 muropeptide ions that were identified using an in silico PG library. The relative abundance for each muropeptide ion was measured by integrating the extracted-ion chromatogram. The percentage of crosslink per PG subunit was measured at 45%. While both 3→3 and 4→3 transpeptide cross-linkages were found in PG dimers, a high abundance of 3→3 linkages was found associated with the trimers. Approximately 43% of disaccharides in the PG of *M. smegmatis* showed modifications by acetylation or deacetylation. A significant number of PG trimers are found with a loss of 41.00 amu that is consistent with *N*-deacetylation, whereas the dimers show a gain of 42.01 amu corresponding to *O*-acetylation of the PG disaccharides. This suggests a possible role of PG acetylation in the regulation of cell wall homeostasis in *M. smegmatis*. Collectively, these data report important novel insights into the ultrastructure of mycobacterial PG.

## Introduction

**Figure 1:**
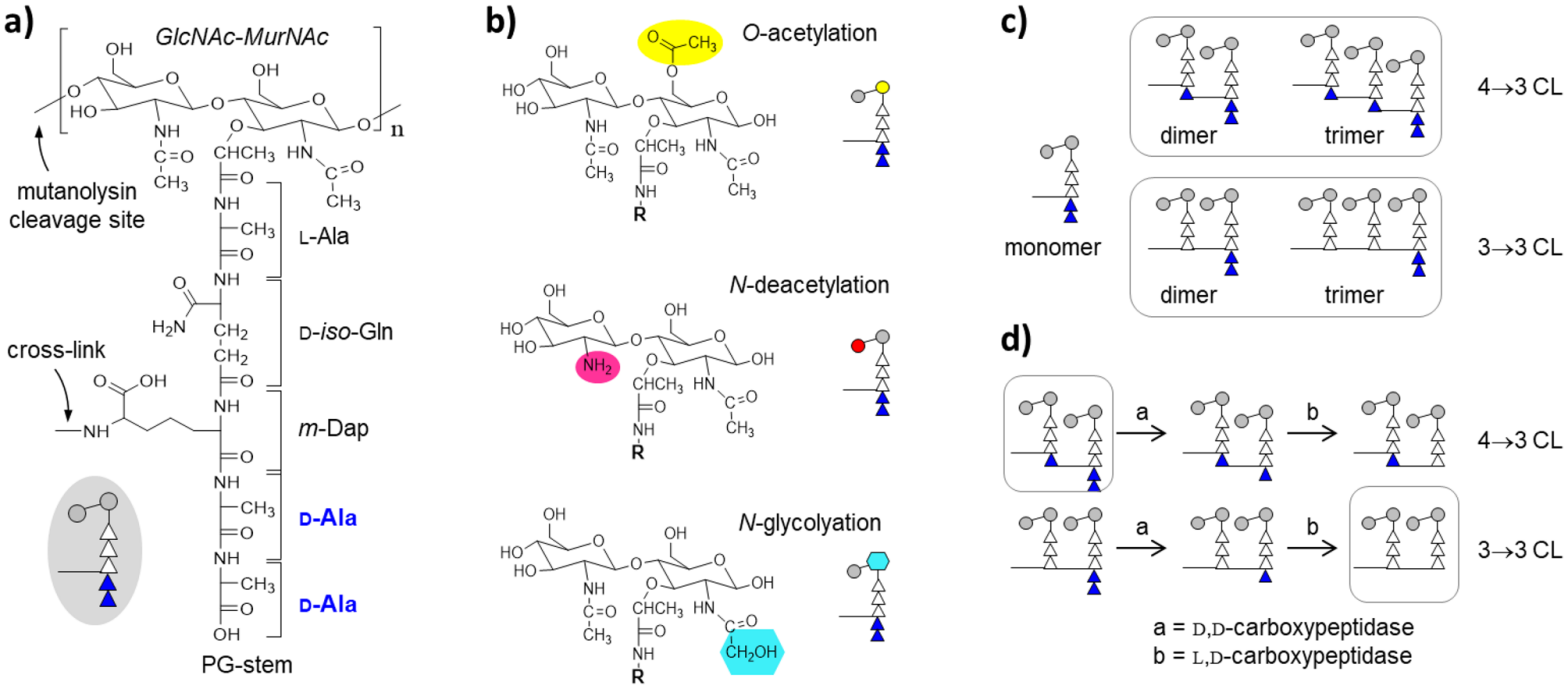
The peptidoglycan of *Mycobacterium smegmatis*. (**a**) Chemical structure of peptidoglycan (PG) repeat unit in *M. smegmatis* (mc2155). The PG-repeat unit consists of a disaccharide *N*-acetyl glucosamine (GlcNAc) and *N*-acetylmuramic acid (MurNAc). A pentapeptide stem unit, L-Ala-D-iso-Gln-*m*-Dap-D-Ala-D-Ala, is attached to the lactic moiety on MurNAc. A schematic representation of a PG-repeat unit is shown as an inset with two grey circles representing the disaccharides, five triangles representing the pentapeptide stem, and two filled triangles representing D-Ala-D-Ala. The muramidase cleave site is indicated by an arrow. (**b**) Chemical modifications of PG disaccharide in *M. smegmatis*: *O*-acetylation, *N*-deacetylation, and *N*-glycolylation. Although *O*-acetylation is shown at MurNAc and *N*-deacetylation at GlcNAc, the actual position at which these modifications occur in mycobacterium has not yet been determined. The R group represents the pentapeptide-stem structure. Schematic representations of disaccharide-modified PG-repeat units are shown on the right with a yellow circle representing *O*-acetylation, a red circle for *N*-deacetylation, and a blue hexagon for *N*-glycolylation. (**c**) Muramidase digestion of isolated cell walls of *M. smegmatis* results in muropeptide fragments that are predominantly monomers, dimers, and trimers. While muramidase digestion cleaves the glycan chains, crosslink which is a peptide bond formed between the *m*-Dap from a PG-stem to the D-Ala of the neighboring stem is preserved. Dimers can have either 4→3 or 3→3 crosslinks. In the 4→3 crosslinks, a peptide bond is formed between the m-DAP to the D-Ala of the neighboring tetrapeptide-stem structure. In the 3→3 crosslinks, a peptide bond is formed between the side chain of m-DAP to the carbonyl carbon on the *m*-DAP of the neighboring tripeptide-stem structure. Trimers have two crosslinks. For illustration, the figure shows two trimers consisting entirely of 4→3 or 3→3 crosslinks. d) PG-stem modifications are carried out by D, D- and L,D- carboxypeptidases which sequentially remove the D-Ala from the uncrosslinked PG-stem structure. Schematic representation of PG-stem modification of dimers with 4→3 and 3→3 crosslinks. The muropeptides without any D-Ala (0 D-Ala) consist of 3→3 crosslinked dimers (bottom right square), whereas the PG dimers with 3 D-Ala are unique to 4→3 crosslinked dimers (top left square). The muropeptides with 1 or 2 D-Ala cannot be unambiguously determined as they can be found in both 3→3 and 4→3 crosslink dimers.

Mycobacteria represent a diverse group of organisms that include etiological agents for serious diseases such as tuberculosis and leprosy. The remarkable adaptability of these organisms and their ability to cause disease in humans has been attributed in part to their unique cell wall structure and composition which plays an important role in withstanding stress, modulating the immune system, and mediating tolerance to drugs^1,2^. The cell wall of mycobacteria has been demonstrated to be integral in the virulence of pathogenic mycobacteria as evidenced by the fact that several drugs used to treat mycobacterial infections inhibit pathways involved in cell wall synthesis^3^. Exterior to the plasma membrane of a mycobacterial cell is surrounded by the robust cell wall structure comprised of peptidoglycan, arabinogalactan, and long-chain mycolic acids, which are collectively known as the mycolyl-arabinogalactan-peptidoglycan complex (mAGP)^4^. The mAGP is comprised of lipids and carbohydrates that are anchored to the peptidoglycan (PG). The PG serves as the cell exoskeleton and a scaffold for mAGP attachment, providing tensile strength necessary for the structural integrity of the cell wall against fluctuating osmotic pressures, and for maintaining the cell shape during growth and division. The monomeric unit that is required for the synthesis of mycobacterial PG comprises of a disaccharide with a peptide stem (Fig. 1a). The disaccharide comprises *N*-acetylglucosamine (GlcNAc) connected to *N*-acetylmuramic acid (MurNAc) by a β-1,4 glycosidic linkage. Each PG-repeat unit has a stem structure with a sequence l-alanyl-γ-d-isoglutamyl-*meso*-diaminopimelate-d-alanyl-d-alanine attached to the lactoyl group of MurNAc (Fig. 1a)^5^.

The chemical composition of PG in bacteria is dynamic, resulting from continuous modifications to its glycan backbone and stem peptide moieties^6^. Such modifications are considered essential for adaptation at different growth phases and alterations to the environment such as changes in nutrients, temperature, and stress from exposure to antibiotics and host immune responses. Some of the known PG modifications in mycobacteria include *N*-glycolylation^7,8^, amidations of the carboxyl groups in diaminopimelate (DAP) and d-Glu^9^, and 1,6-anhydro ring formation of MurNAc^10^. Disaccharide modifications including *O*-acetylation of MurNAc^11^ and *N*-deacetylation of GlcNAc^12^ have not been reported in mycobacterial PG^3^. The *N*-glycolylation of the PG MurNAc (Fig. 1b) is a common feature found in mycobacteria and other closely related *Actinomycetales*^7,13^. *N*-glycolylation was first identified in *M. smegmatis*^14^ where the MurNAc in Park’s nucleotide (cytoplasmic PG precursor) is oxidized to *N*-glycolylmuramic acid (MurNGlyc)^15^ by *N*-acetyl muramic acid hydroxylase (NamH)^7^ in the cytoplasm. The only exception is *Mycobacterium leprae* where the MurNAc is found to be exclusively *N*-acetylated^16^. The function of *N*-glycolylation is unknown, but an increase in PG glycolylation of *M. smegmatis* following exposure to cell wall-targeting antibiotics suggests that the modification may contribute to increased stabilization of the PG-lattice structure^8^. In addition, the *N*-glycolylation of PG is also thought to increase resistance to lysozyme degradation^7^.

The final assembly of PG involves two essential enzymatic processes: transglycosylation, and transpeptidation^17^. Transglycosylation comprises the polymerization of the PG subunits into a two-dimensional growing strand of glycan chain, and transpeptidation entails crosslinking of the peptide chains by forming a transpeptide bond between the peptide stems to generate a three-dimensional meshed lattice structure. The stem peptides are crosslinked by two chemically distinct linkages, either a 4→3 or a 3→3 transpeptide bond (Fig. 1c). 4→3 linkages are generated by d,d-transpeptidases, also known as penicillin-binding proteins (PBPs), by forming a transpeptide bond between d-Ala at the fourth position of the donor stem and the third amino acid of the acceptor stem which is meso-diaminopimelic acids (m-Daps) in *M. smegmatis*. Concomitant with the 4→3 crosslink formation, the terminal d-Ala at the fifth position of the donor stem is cleaved (Fig. 1d). In most eubacteria, the PG is crosslinked predominantly with 4→3 linkages. For example, the PG of *Staphylococcus aureus* is exclusively 4→3 crosslinked^18^. For the 3→3 crosslinks, l,d-transpeptidases (LDTs) catalyze the transpeptide bond formed between two amino acids at the third position of the donor and acceptor stem peptides^19–21^. The PG that is predominantly 3→3 crosslinked is considered atypical as the historical model of PG only included 4→3 linkages^22^. In addition to mycobacterial species, the PG of *Clostridioides difficile* predominantly consists of 3→3 linkages, whereas in the case of *E. faecium*, and *E. coli*, atypical type is found only in low abundance^20,23–26^. In *Mycobacterium tuberculosis,* slow-growing mycobacteria whose infection causes tuberculosis, approximately 67–80% of PG crosslinks are of the 3→3 type^20,27,28^. In *Mycobacterium abscessus*, rapidly growing mycobacteria, 3→3 linkages also predominate the PG crosslinks^23^. This suggests that the LDTs play a critical role in maintaining the cell wall integrity^29^ and cell shape^30^ in mycobacteria^31^. The significant role of d,d-transpeptidases and LDTs in the viability of mycobacteria has been demonstrated via the potent activity of the carbapenem class of β-lactams inhibiting the activities of these enzymes^19,27,28,30,32^.

In this study, we elaborate on the molecular structure of PG in mycobacteria through a detailed compositional analysis of the PG of *M. smegmatis* using ultra-performance liquid chromatography-mass spectrometry (LC–MS)^10,18,23,33–40^. Although *M. smegmatis* is commonly used as a surrogate for *M. tuberculosis* studies, recent reclassification of *M. smegmatis* to a novel genus *Mycolicibacterium* suggests limitation as a surrogate for *M. tuberculosis*^41^. Nonetheless, an in-depth analysis of *M. smegmatis* PG composition and its chemical modifications by LC–MS is essential to understanding the fundamental biology of mycobacterial cell wall biosynthesis and will provide insights into the unique chemical composition and architecture of mycobacterial PG.

## Results and discussion

### Accurate PG quantification by LC–MS

PG composition analysis was performed on isolated cell walls of *M. smegmatis* (wildtype strain mc^2^155) that were grown in 500 ml of supplemented 7H9 broth harvested at stationary phase with an optical density of 1.6 at 600 nm (OD_600_, 1.6). Since PG composition is highly dynamic in response to changes in growth conditions, the stationary phase was selected for the analysis of mature PG. In the stationary phase, as nutrients deplete, bacteria cease to grow and adopt a uniform cell morphology. In contrast, bacteria during the mid-exponential growth phase show a mixture of cells at various stages of cell division. Hence, isolated cell walls from bacteria during the mid-exponential phase consist of PGs from the dividing septum, expanding poles, immature sidewalls, and mature sidewalls, giving rise to a more complex PG composition. Typically, the isolated cell walls are treated with hydrofluoric acid to remove covalently bonded non-PG biopolymers that are attached to the PG prior to the digestion by mutanolysin, an *N*-acetylmuramidase, to cleave the β-1,4 glycosidic bond between MurNAc and GlcNAc of the PG (Fig. 1a). The resulting solubilized muropeptide fragments are then separated using liquid chromatography (LC) and detected using high-resolution mass spectrometry (MS). The use of acid hydrolysis significantly improves the chromatographic resolution and separation of muropeptides by removing the PG acetylation and thereby reducing the chemical diversity of muropeptide fragments. This is crucial when using conventional spectral absorption methods for accurate quantification which relies on the absorption integral of the elutants during the separation. For our approach, however, acid hydrolysis of cell walls was omitted to preserve PG O-acetylation which led to a complex mixture of muropeptides following the mutanolysin digestion. The complex mixture resulted in multiple muropeptides co-eluting during the LC separation.

The muropeptides from the mixture were identified by matching the observed accurate m/z values from LC–MS to a muropeptide mass library of calculated m/z generated using MATLAB (MathWorks). The library included all possible combinatorial PG modifications consisting of 2234 possible muropeptide ions. The abundance of each muropeptide species was measured by integrating the extracted-ion chromatogram (XIC) of the selected ion across all its isotopic distribution. Then the relative abundance of each muropeptide was calculated by dividing its integrated XIC sum by the total integral sum of all muropeptides. Unlike muropeptide quantification by UV absorption which requires chromatically resolved peaks, the integral of XIC is not affected by the overlapping co-elution of muropeptides as each muropeptide with a unique m/z value is resolved in the mass–charge dimension. Of 2234 unique muropeptide ions that were considered, we identified and quantified 130 muropeptide ions. All LC–MS measurements were carried out in triplicate using mutanolysin-digested isolated cell walls prepared from a single 500-ml growth culture. Hence, the calculated standard deviation for technical replicate with an error associated with the precision of measurement, but not biological variance as biological triplicates were not pursued in the interest of method validation. In our analysis, the calculated 95% confidence intervals were narrow, ranging from 0.01 to 2% for abundant muropeptide species, and 2 to 5.7% for the less abundant species.

### PG crosslinking in M. smegmatis

Isolated cell walls were not subjected to acid hydrolysis to preserve PG *O*-acetylation. Although this does not affect the PG crosslinking, without acid hydrolysis, PGs that are covalently attached to arabinogalactan were excluded from the analysis. From a total of 130 muropeptide ions that were identified, 29 were monomers, 71 were dimers, and 30 were trimers. The abundance of each muropeptide was quantified by adding the integral of the XIC of selected ions^42^. The measured ion intensities were multiplied with a factor, *c* + 1, where *c* is the number of crosslinks found in the muropeptide fragment to account for the number of PG-repeat units found in each muropeptide fragment. For example, the integrated ion intensity of monomer (*c* = 0) is multiplied by one, a dimer (*c* = 1) is multiplied by a factor of two, and a trimer (*c* = 2) by three to yield relative intensities which are proportionate to the total number of PG-repeat units found for each muropeptide species. This data was then assimilated to generate the macromolecular composition of the corresponding PG found in mature cell walls.

**Figure 2:**
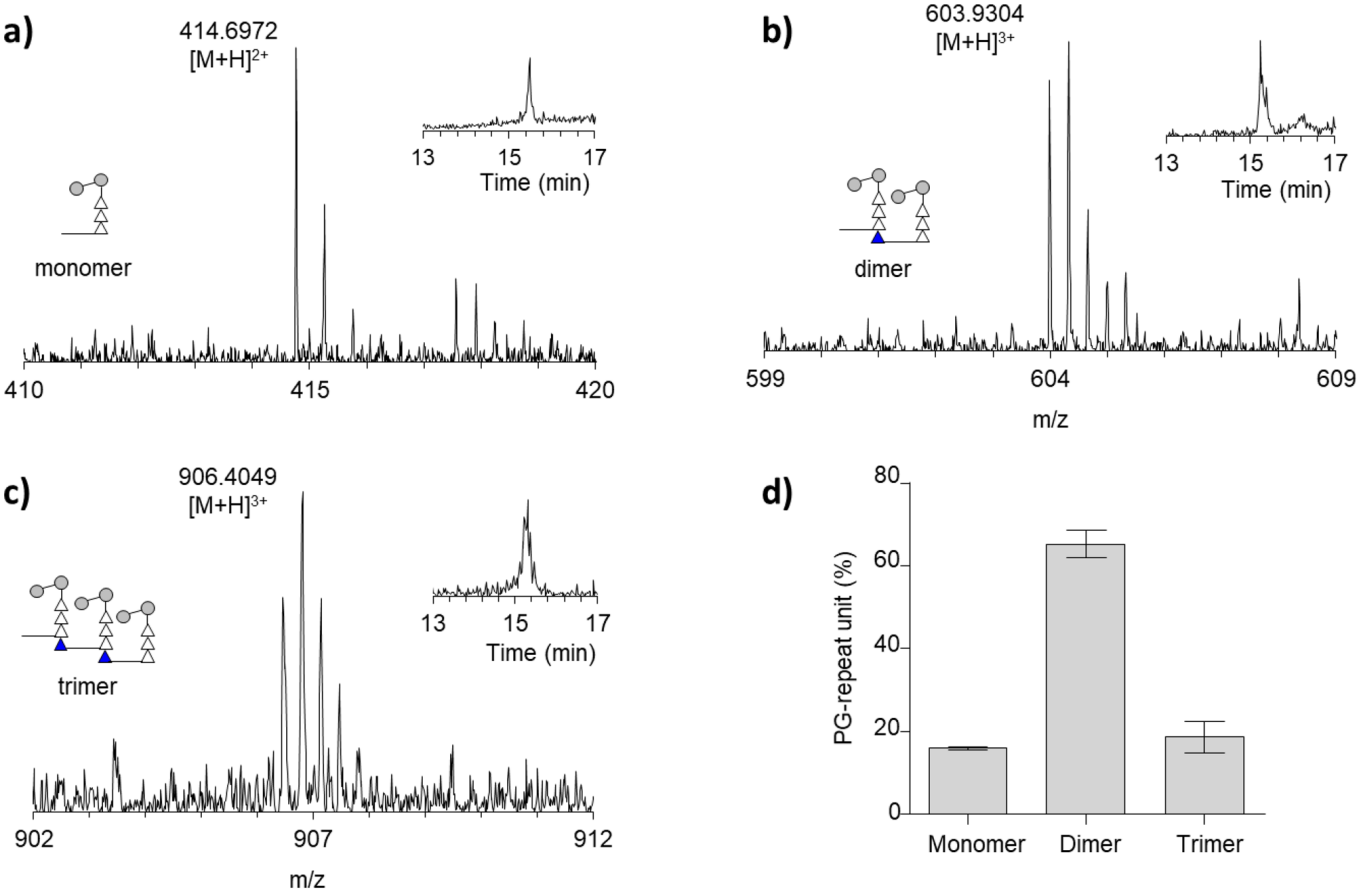
PG crosslinking in *M. smegmatis*. (**a**–**c**) Mass spectra of the monomer, dimer, and trimer from the digestion of cell wall peptidoglycan of mc2155 with the extracted ion chromatogram (XIC) as an inset (right). The schematic representation of the corresponding muropeptide fragments is shown as figure inset (left) and excludes any modification on the muropeptides except for the highlighted modification. (**d**) Profile of the muropeptide fragments as a function of the number of repeat units. Calculated PG crosslinking efficiency for mc2155 strain of *M. smegmatis* is 45.11 ± 0.79, and the average fragment size of the muropeptide is 1.82 ± 0.03 PG-repeat units. All error bar represents a 95% confidence interval (*n* = *3*) for technical error. The composition is shown in Table S1. The chemical structure of the representative MS with various modifications is shown in Fig. S1.

The representative LC–MS spectra of muropeptide fragments with an increasing number of crosslinks; monomer, dimer, and trimer are shown in Fig. 2a–c. Each MS spectrum is shown with a schematic representation of the muropeptide fragment and the XIC for the selected m/z value. Figure 2d shows the profile of muropeptides in *M. smegmatis* based on the number of crosslinks. The predominant muropeptide species were PG dimers (65.26 ± 3.41%), with a comparable number of trimers (18.72 ± 3.74%), and then monomers (16.03 ± 0.35%) as a proportion of the total number of PG-repeat units (Fig. 2d). The absence of muropeptides that were greater than trimers in mutanolysin-digested cell walls of *M. smegmatis* suggests a low degree of PG crosslinking^10,40^ compared to *S. aureus* which has oligomers that are large as nonamers and greater have been reported^18,36,42^. The PG crosslinking efficiency^43^, which is defined as the percentage of crosslink per PG subunit using a method described by Chang et al.^18^, for the mutanolysin-digested isolated cell walls of *M. smegmatis* was 45.11 ± 0.79%. The average muropeptide fragment size was calculated by multiplying the normalized XIC intensities for each muropeptide ion by the number of PG-repeat units in the muropeptide that are associated with the modification. The fragment size is presented as the number of PG-repeat units. For mutanolysin-digested PG, the average muropeptide fragment size was 2.03 ± 0.04 PG-repeat units for *M. smegmatis* at a stationary phase.

### PG-stem alanylation and crosslinking in M. smegmatis

**Figure 3:**
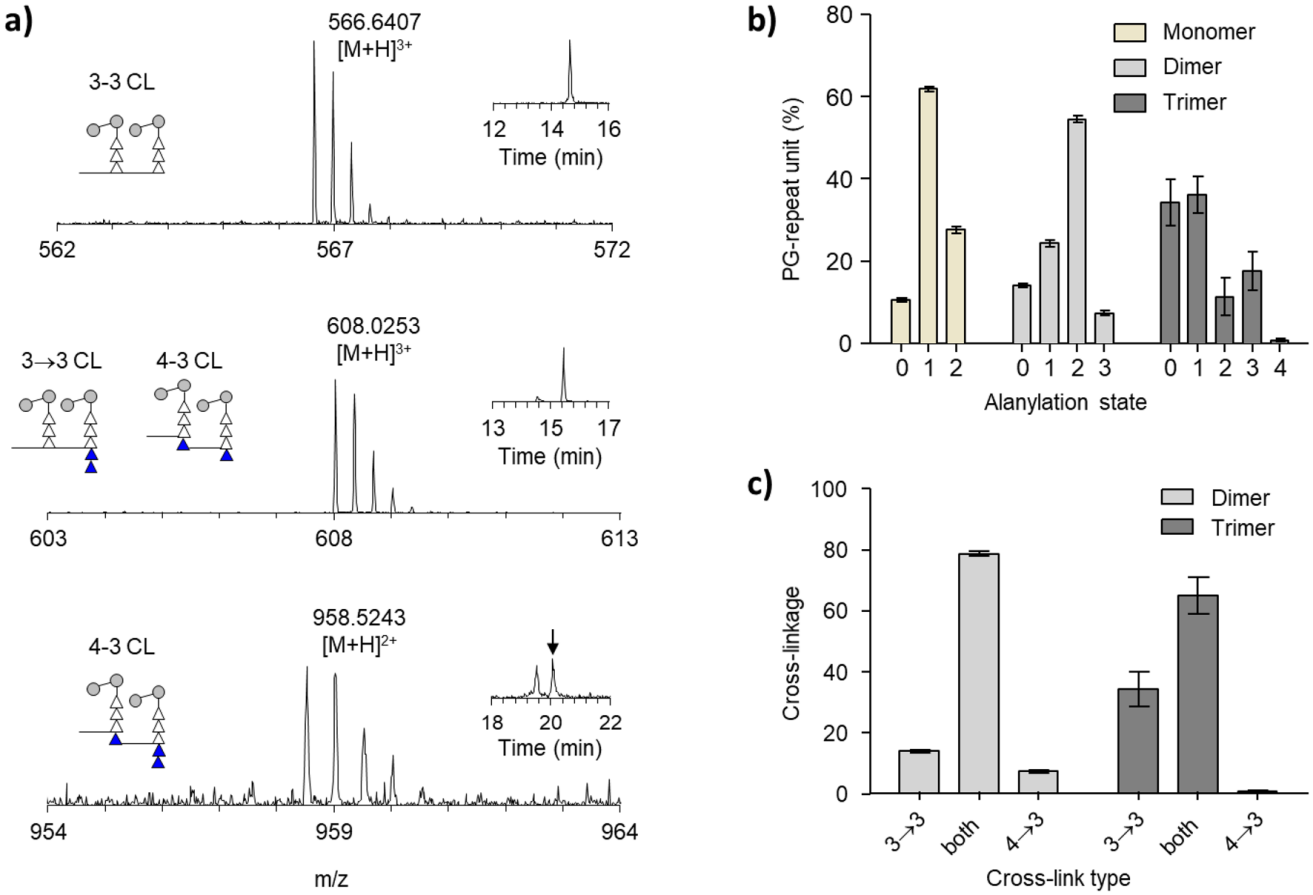
D-Alanylation and PG crosslinking from the mutanolysin digested cell wall of *M. smegmatis*. (**a**) Representative mass spectra of PG dimers with varying PG-stem lengths with the corresponding XICs and schematic diagram of PGs shown as figure insets. This schematic diagram excludes any modification on the muropeptides except for the highlighted modification. The chemical structures of the representative MS are shown in Fig. S2. Muropeptide dimers without D-Ala in the PG-stems can be unambiguously assigned to 3→3 crosslinks (top), but dimers with two D-Ala can be assigned to either 3→3 or 4→3 crosslinks (middle). In contrast, dimers with three D-Ala are unambiguously assigned to a 4→3 crosslink (bottom). (**b)** Proportions of the PG oligomers are categorized based on the net number of D-Ala in the PG-stem structure of the observed muropeptide fragment. The PG-stem structure for monomers with an alanylation state of zero has a tripeptide stem, an alanylation state of one has a tetrapeptide, and two has a pentapeptide-stem structure. The composition is shown in Table S3. (**c)** Quantification of 4→3 and 3→3 crosslinks in dimers and trimers based on the number of D-Ala in the PG-stem structure. In dimers, a larger proportion of the PG fragment containing one or two D-Ala can be assigned to either 3→3 or 4→3 crosslinks, however, a small fraction of muropeptides that have zero or three D-Ala can be unambiguously assigned respectively to 3→3 and 4→3 crosslinks. In trimers, each muropeptide fragments have two crosslinks. The intensities of the trimers with combination crosslinks of 3→3 and 4→3 are summed and represented in the bar graph as “both”. The summed intensities of trimers containing exclusively 3→3 or 4→3 crosslinks are shown in the bar graph as “3→3” and “4→3”, respectively. The composition is shown in Table S4. All error bar represents a 95% confidence interval (*n* = *3*) for technical error.

A PG dimer is comprised of two PG subunits with its peptide side chains crosslinked. The LDTs in *M. tuberculosis*^19,20^ and *E. faecalis*^20,21^ that catalyzes 3→3 crosslinks have specificity for the tetrapeptide-stem acyl donor as a substrate, while the PBPs for 4→3 crosslink have specificity for the pentapeptide-stem acyl donor. Although the substrate specificity of LDTs and PBPs in *M. smegmatis* have not been characterized, they are likely to be specific to tetrapeptide-stem and pentapeptide-stem, respectively. Hence, PG dimers of *M. smegmatis*, depending on the cross-linkage type, the number of amino acids in the acyl-donor stem is fixed (Supporting Table S2). For example, in a 3→3 crosslinked dimer the acyl-donor stem is a tripeptide that lacks the fourth and terminal d-Ala residues found in the pentapeptide substrate, whereas in a 4→3 crosslink dimer, the acyl-donor stem is a tetrapeptide with d-Ala as the fourth residue (Fig. 1d). In the acyl-acceptor stem of the dimer, the total number of d-Ala can also vary from zero to two due to PG-stem modifications by d,d- and l,d- carboxypeptidases that catalyze a sequential removal of the terminal d-Ala (Fig. 1d). Representative mass spectra of PG dimers with varying numbers of d-Ala are shown in Fig. 3a. The minimum number of d-Ala possible in a PG dimer is zero and this is specific to 3→3 crosslink (Fig. 3a, top). In contrast, the maximum number of d-Ala possible in a dimer is three, and this is specific to the 4→3 crosslink (Fig. 3a, bottom). Any PG dimer containing one or two d-Ala assigned to either 3→3 or 4→3 crosslinks (Fig. 3a, middle).

To determine the relative quantities of 3→3 and 4→3 crosslinks in the cell walls of *M. smegmatis*, the observed muropeptide ion intensities, independent of disaccharide modifications, were added according to their classification solely based on the number of crosslinks (monomers, dimers, and trimers) and its alanylation state. The d-alanylation state refers to the total number of d-Ala found on the muropeptide fragment. The ion intensities of muropeptides were multiplied by the total number of PG-repeat units found in each fragment. The normalized intensities of muropeptides were then grouped based on the number of crosslinks and subcategorized based on their alanylation state. Each group is normalized to 100 percent and the relative portions of their alanylation state are shown as a bar graph in Fig. 3b.

The alanylation pattern of the PG monomers (Fig. 3b, left) reveals that the corresponding stem structure predominantly terminated in a tetrapeptide (61.81 ± 0.48%), followed by pentapeptide (27.62 ± 0.89%), and tripeptide (10.57 ± 0.46%). Since monomers are likely to be found in the regions of cell walls with reduced crosslinking, the high abundance of tetrapeptide monomers suggests that d,d-carboxypeptidases are highly active in the regions of cell walls with reduced crosslinking. The relatively low abundance of tripeptide monomers suggests that the activity of l,d-carboxypeptidases that generate these fragments is required at reduced levels in the newly built cell wall. An alternate source of tripeptide monomers is d,d-endopeptidases that cleave 4→3 cross-linkages to generate substrates for the LDTs to form 3→3 crosslinks.

The alanylation of the PG dimers (Fig. 3b, middle) shows that the muropeptides with zero d-Ala can be unambiguously assigned to 3→3 crosslinked dimers that constituted 13.95 ± 0.48% of all the dimers found in the cell wall. The dimers containing one and two d-Ala cannot be unambiguously assigned to either 3→3 or 4→3 crosslinks (Supporting Table S2), and altogether they constituted the largest fraction measured at 78.69 ± 0.83%. The dimers with three d-Ala which are specific to a 4→3 cross-linkages were only observed at 7.35 ± 0.53% (Fig. 3c, left). For dimers, the ratio of muropeptides with unambiguously assigned 3→3 to 4→3 crosslinks was approximately 2:1.

In PG trimers, there are two crosslinks between the PG subunits (Supporting Table S2). The first crosslink connects the first and second stem peptides, and the second crosslink connects the second and third stem peptides. We observed that a large fraction of the PG trimers (65.02 ± 6.00%) possessed one to three d-Ala (Fig. 3b, right). These muropeptides could be assigned to trimers with two consecutive 3→3 or 4→3 crosslinks or mixed with having each of both 3→3 and 4→3 crosslinks (Fig. 3c, right). In contrast, 34.26 ± 5.69% of the trimers without any d-Ala, as in the case of dimers, could be unambiguously assigned to muropeptides with two consecutive 3→3 crosslinks. Likewise, the trimers with four d-Ala were also unambiguously assigned to muropeptides with two consecutive 4→3 crosslinks. The trimer with two consecutive 4→3 crosslinks comprised less than one percent (0.73 ± 0.31%) of the total trimers detected (Fig. 3d). The ratio of trimers with two consecutive 3→3 crosslinks to the trimers with two consecutive 4→3 crosslinks is approximately 47:1, which is a significant increase in comparison to dimers where the ratio was 2:1. This suggests that the regions of highly crosslinked cell walls that contain trimers are tentatively 3→3 crosslinked; whereas the regions of cell walls with a lower degree of crosslinking consist of a mixture of both 4→3 and 3→3 linkage types.

### N-glycolylated muramic acid in PG of M. smegmatis

**Figure 4:**
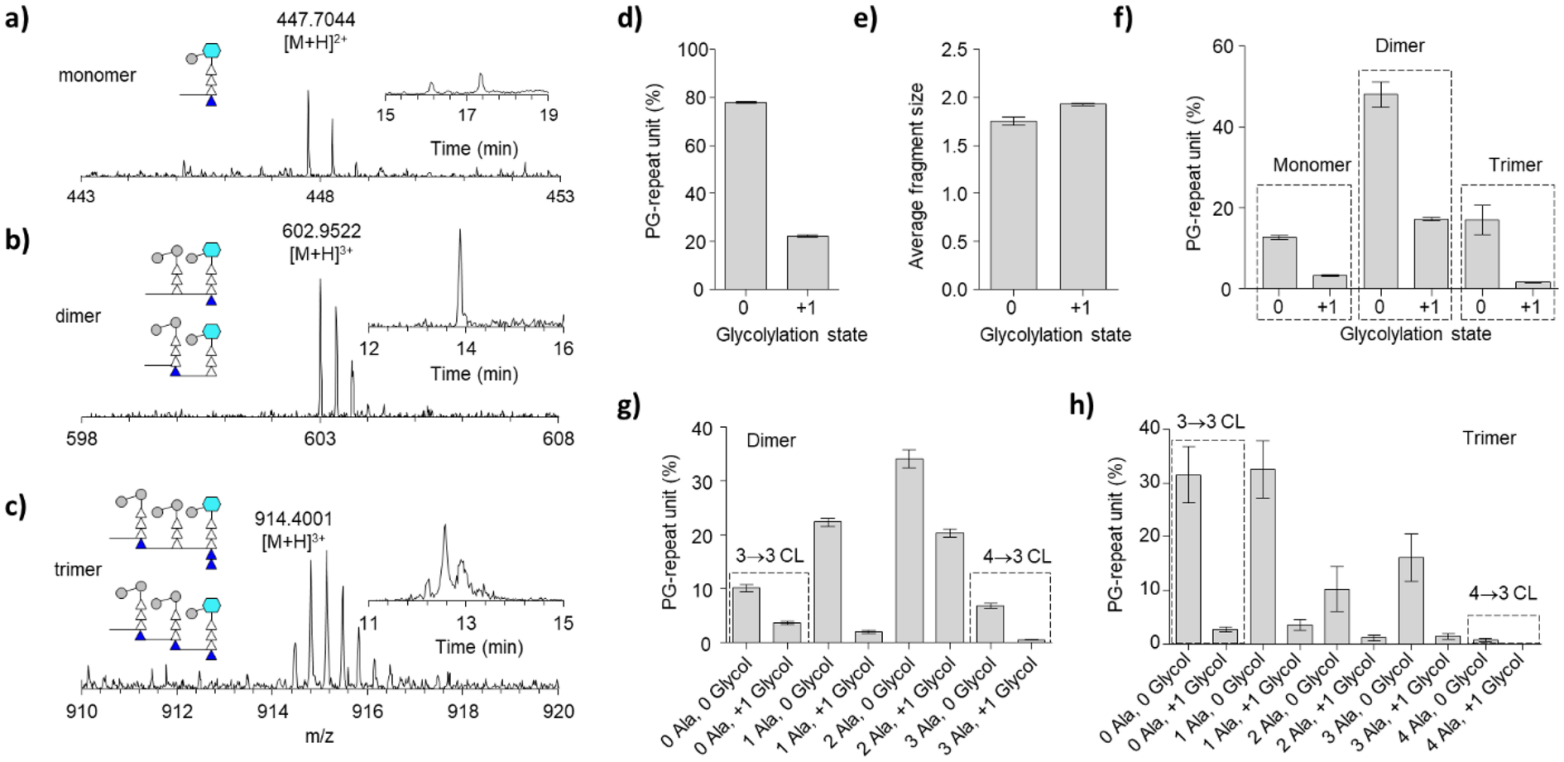
*N*-glycolylated PG of *M. smegmatis*. (**a**–**c**) Mass spectra of PG monomer, dimer, and trimer with *N*-glycolylated muramic acid (MurNGlyc) and peptide stem length terminating in a tetrapeptide, a tripeptide, and a pentapeptide structure. Insets show the schematic representation of MurNGlyc, shown as a blue hexagon, with the XIC for the proposed muropeptide structure. Although the number of MurNGlyc in a muropeptide can be inferred from the accurate mass, the position of MurNGlyc in dimers and trimers was not determined. The cartoon representation excludes any other PG variation than N-glycolylation of muramic acid. The chemical structures of the representative MS are shown in Fig. S3. The glycolylation state of ‘0’ indicates the muropeptide fragments without MurNAc modification and ‘ + 1’ for the muropeptide unit with a maximum of one MurNGlyc moiety. (**d)** The relative abundances of muropeptides with variation in the muramic acid moiety of a disaccharide unit as a function of its glycolylated state. The relative abundance was determined by summing the integrals of the XIC ion current of each muropeptide. Prior to the summing, each XIC ion current was normalized by scaling the intensity with respect to the total number of PG-repeat units found in each fragment. (**e)** Calculated average fragment size based on glycolylation state. (**f)** The composition of PG *N*-glycolylation is categorized based on oligomerization (see Table S7). Approximately 26% of the dimers are *N*-glycolylated, followed by 21% of monomers, and 9% of trimers. (**g)** The *N*-glycolylation in PG dimers based on the 3→3 and 3–4 CL types. (**h)** The *N*-glycolylation in PG trimers based on the 3→3 and 3–4 CL types. All error bars represent a 95% confidence interval (*n* = *3*) for technical error.

The *N*-glycolylated muramic acid (MurNGlyc) is an exclusive modification found in the PG of *Mycobacterium spp*^6^. The function of PG *N*-glycolylation remains unclear as a *namH* deletion mutant, which was defective for *N*-glycolylation, does not show a significant change in cell morphology or growth kinetics^44^. Nonetheless, this modification is believed to contribute to lysozyme and β-lactam resistance^6,7^ and is thought to increase the overall stability of the cell wall^6,45^. Figure 4a–c shows the positive ion mass spectrum of the PG monomer, dimer, and trimer from the mutanolysin digested cell walls of *M. smegmatis* with a glycolylation state of ‘+1’ which indicates that the muropeptide fragment contains one MurNGlyc. The corresponding XIC with a schematic representation of PG is shown as a Figure inset. The mature cell walls of *M. smegmatis* show a mixed presence of PG-repeat units with both MurNAc and MurNGlyc^8^. The relative ratio of PG-repeat units containing MurNGlyc to MurNAc was approximately 2:7 (Fig. 4d) with the abundance of PG-repeat units containing a MurNGlyc measured at 22.22 ± 0.45% and MurNAc at 77.78 ± 0.45% (Table S5).

The calculated average muropeptide fragment size without *N*-glycolylation was 1.75 ± 0.04 PG-repeat units, and the muropeptide fragment with MurNGly was 1.93 ± 0.01 (Fig. 4e, Table S6). Figure 4f shows the distribution of muropeptides with MurNGlyc modification based on the oligomerization state. Individual percentile composition of the muropeptide with and without MurNGlyc modification is included in the Supporting Information Table S7. The dimers constituted the largest fraction, representing approximately 65% of all PG-repeat units, of which 26% of dimers possess MurNGlyc modification. The trimers constituted the second-largest fraction with approximately 19% of all PG-repeat units of which 9% possessed MurNGlyc modification. Finally, monomers constituted the smallest fraction, representing 16% of the total PG subunits of which 21% possess the MurNGlyc modification. *N*-glycolylation is not uniformly distributed, but it is preferentially found in the PG dimers and monomers with approximately two to three folds higher in abundance than in trimers. The fraction of trimers with a MurNGlyc modification was small, constituting less than 2% of the total PG-repeat units. Hence, this suggests that *N*-glycolylation of PG is more commonly found in regions of cell walls with reduced crosslinking with greater frequency in 3→3 than 4→3 crosslinked muropeptides for both dimers (Fig. 4g) and trimers (Fig. 4h).

### PG-disaccharide acetylation in M. smegmatis

The PG disaccharide is commonly modified by *O*-acetylation of MurNAc at C-6 hydroxyl moiety and *N*-deacetylation of GlcNAc at the C-2 position (Fig. 1b) in other bacteria^3,46^, but in *Mycobacterium*, such modifications have not been reported. A proposed *N*-deacetylase (Rv1096) from *M. tuberculosis* has been shown to exhibit deacetylase activity on isolated PG of *M. smegmatis*^47^. The *N*-deacetylase activity is thought to shield the pathogen from host immune surveillance by preventing the host pattern recognition receptors from binding to the PG and thereby increasing the pathogen’s survival^48^. To monitor the PG *O*-acetylation in *M. smegmatis*, the acid treatment of isolated cell walls was omitted. This is because the *O*-acetyl group is highly labile under acidic and basic conditions, and the use of strong acids, such as hydrofluoric acid, readily removes *O*-acetyl groups from the PG. Without acid hydrolysis, which is necessary for removing arabinogalactan from cell walls, the PG that are covalently attached to the glycopolymers are excluded from the analysis.

**Figure 5:**
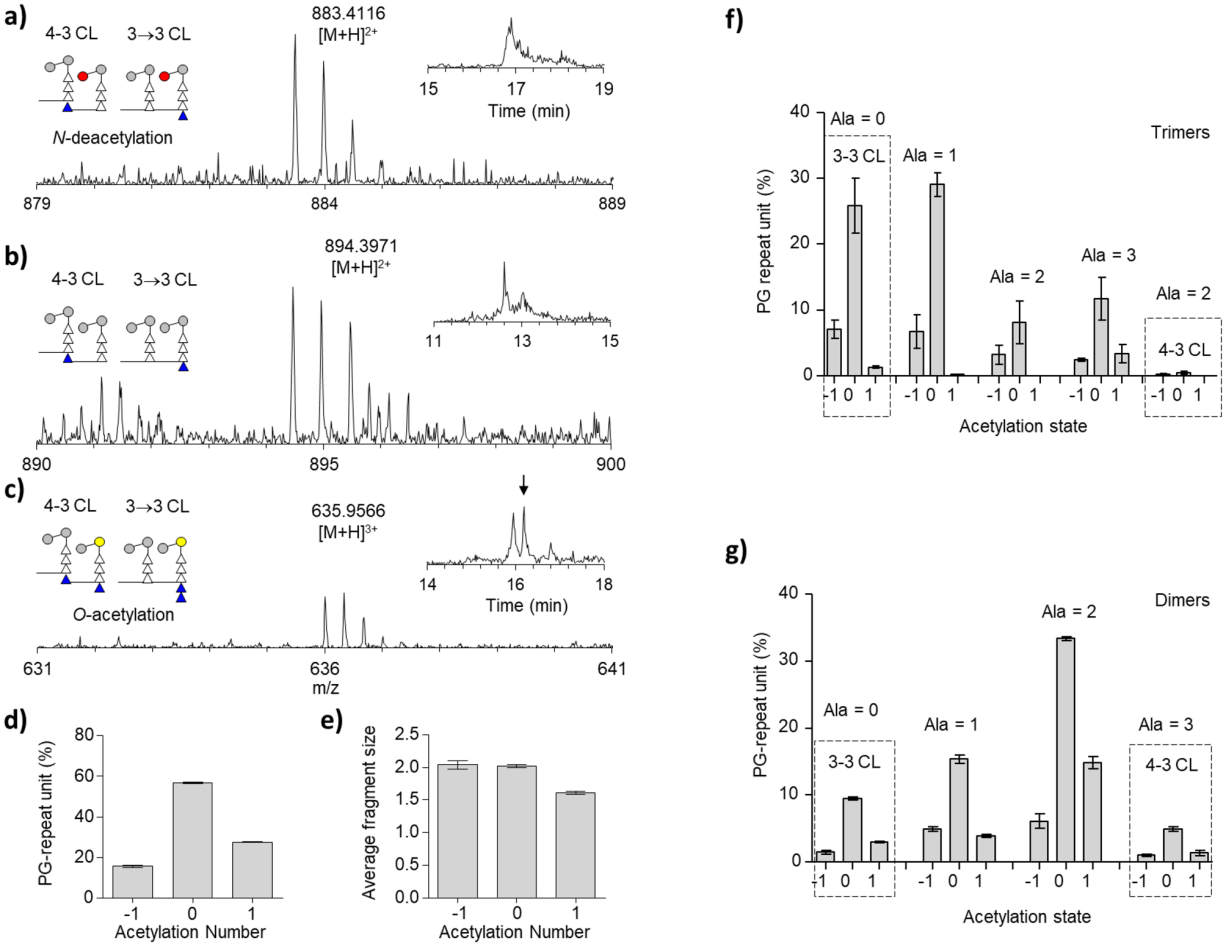
Peptidoglycan acetylation of mc2155 cell walls. The PG disaccharide repeat unit, GlcNAc-MurNAc can be chemically modified by *O*-acetylation of MurNAc (+ 1) or *N*-deacetylation of GlcNAc (-1). (**a–c)** Mass spectra, XIC, and schematic representation of charged PG dimeric unit with acetylation states ranging from − 1 to + 1. Insets show a schematic representation of *N*-deacetylated and *O*-acetylated glycan as red and yellow-colored circles, respectively. The blue triangles represent D-Ala. Since the positions for *O*-acetylation and *N*-deacetylation have not been determined, the modification could have occurred in either GlcNAc or MurNAc. The cartoon representation excludes any other PG variation than net acetylation. The chemical structures of the representative MS are shown in Fig. S4. (**d)** Breakdown of each PG ion observed according to the number of acetylation states. The unmodified muropeptides fragments are found at 56.72 ± 0.49%, followed by *O*-acetylated muropeptides at 27.60 ± 0.25%, and *N*-deacetylated at 15.67 ± 0.47%. The composition is shown in Table S8. (**e)** Calculated average fragment size in muropeptide as a function of acetylation. (**f)** Degree of crosslinks in PG trimers calculated based on net acetylation. The composition is shown in Table S9. (**g)** Degree of crosslinks in PG dimers calculated based on net acetylation. The composition is provided in Table S12. All error bar represents a 95% confidence interval (*n* = *3*) for technical error.

Our LC–MS analysis of mutanolysin-digested cell walls of *M. smegmatis* confirmed the presence of muropeptides with a gain of 42.01 or a loss of 41.00 amu from the unmodified PG which was consistent with PG modification by O-acetylation and N-deacetylation, respectively. Figure 5a–c shows the representative mass spectra of PG dimers from mutanolysin-digested isolated cell walls of *M. smegmatis* with increasing order of the acetylation state. The schematic representation of the PG dimers and the corresponding XIC of the selected ion is shown in the Figure inset. The acetylation state of “− 1” is assigned to muropeptides with an *N*-deacetylation (Fig. 5a, red circle), the state of “0” to muropeptides with unmodified disaccharide (Fig. 5b), and the state of “+1” to muropeptides with an *O*-acetylation (Fig. 5c, yellow circle). Although the schematic figures are shown with *N*-deacetylation of GlcNAc and *O*-acetylation of MurNAc, the positions at which the acetyl groups are either added or removed from the disaccharide were not determined. Therefore, the schematic shown in Fig. 5 and the chemical structure shown in Fig. S4 correspond to one of several possible structures that only differ by the position of acetylation and deacetylation. The PG composition analysis was performed by classifying the intensities of all 130 distinct ions of monomers, dimers, and trimers according to their acetylation state. The analysis shows that 27.60 ± 0.25% of PG-repeat units have *O*-acetylation and 15.67 ± 0.47% with *N*-deacetylation of PG disaccharide (Fig. 5d). Combined, approximately 43% of all the PG-repeat units in the cell walls of *M. smegmatis* have disaccharide modification by either *N*-deacetylation or *O*-acetylation (Table S8).

To understand where the PG *N*-deacetylation and *O*-acetylation modifications occur in the cell wall, an average fragment size of muropeptide for each modification was calculated (Fig. 5e). For *N*-deacetylation, the average fragment size for the muropeptides was 2.04 ± 0.06 PG-repeat units, and for *O*-acetylation the number was 1.61 ± 0.03. Since the numbers “1”, “2”, and “3” correspond to PG monomer, dimer, and trimer, respectively. To further characterize the occurrences of *N*-deacetylation and *O*-acetylation in the muropeptides, the trimers and dimers were subcategorized based on their alanylation, crosslinking, and acetylation states. Figures 5f show the breakdown of the trimer in percentile PG-repeat units. The dotted box in Fig. 5f (left) shows the bar graph of trimer composition with an alanylation state of “0” which corresponds to trimers having two consecutive 3→3 cross-linkages. This group was further subcategorized based on their acetylation states “− 1”, “0”, and “1” (shown on the x-axis). The composition of trimers in their acetylation states expressed in the percent PG-repeat unit is provided in the Supporting Table S9. The percentage of 3→3 and 4→3 crosslinked trimers with a disaccharide modification are shown in Table S10 and S11, respectively.

For trimers, the percent PG-repeat unit compositions of trimers in their acetylation states are provided in the Supporting Table S9. Independent of crosslinkage type, approximately 19.7% of trimers are found to have one *N*-deacetylation, 4.9% with one *O*-acetylation, and the remaining 75.3% with unmodified disaccharides. The composition is further subcategorized based on the linkage type. For trimers with two consecutive 3→3 crosslinkages, approximately 21% have one *N*-deacetylation, 4% with an *O*-acetylation, and the remaining 67% with unmodified disaccharides (Table S10). For trimers with two consecutive 4→3 crosslinkages, the fraction of disaccharide *N*-deacetylation is also high at 33%, but interestingly *O*-acetylation of trimers having two consecutive 4→3 crosslinkages was not observed (Table S11). This indicated that the PG disaccharide of PG trimers, which are predominantly 3→3 crosslinked (Fig. 3c), are found preferentially with *N*-deacetylation (19.7%) over *O*-acetylation (4.9%).

For PG dimers, the percent PG-repeat unit compositions of dimers in their acetylation states are provided in the Supporting Table S12. Accounting for all dimers, we found that approximately 13.7% of all dimers have an *N*-deacetylation, 23.1% with an *O*-acetylation, and the remaining 63.2% with unmodified disaccharides. Further analysis of dimers, the percentage of *N*-deacetylation and *O*-acetylation of disaccharides based on crosslinkage types 3→3 and 4→3 are shown in Tables S13 and S14, respectively. Although a significant portion of the observed dimers could not be unambiguously assigned to a specific crosslinkage type, of the ones that were assigned, a greater number of PG dimers were found with *O*-acetylation than N-deacetylation for both types of crosslinkage. In general, PG dimers are found with a greater frequency of having an *O*-acetylation of PG disaccharide (Fig. 5g). In contrast, trimers show a greater frequency of *N*-deacetylation (19.7%) over O-acetylation (4.9%). This difference in the degree of PG acetylation based on the degree of crosslink and crosslinkage type suggests an interesting possibility that PG acetylation may play an important role in the spatial and temporal regulations of cell wall maturation and modifications in *M. smegmatis*.

### PG composition and lattice structure

The cell wall biosynthesis in *M. smegmatis* differs from other rod-shaped bacteria such as *E. coli* and *B. subtilis* where the nascent PG is inserted uniformly throughout the lateral sidewalls with the polar caps remaining metabolically inactive^49,50^. In *M. smegmatis*, the nascent PG biosynthesis occurs at the poles^31,51^ by PBPs. This has been visualized using fluorescent-labeled vancomycin which binds to the d-Ala- d-Ala of the uncrosslinked PG stem found in the nascent PG^52^. A model of PG maturation proposed by Baranowski et al.^31^, shows that the new growth at the poles carried out by PBPs is remodeled as the cell elongates by d,d-endopeptidases in concert with LDTs where d,d-endopeptidases cleave 4→3 crosslinks to provide the tetrapeptide-stem substrate for the LDTs which in turn generate 3→3 crosslinks. The deletion of LDTs results in the deformation and blebbing of the lateral sidewall^31^. Hence, the LDTs are thought to be aligned along the lateral sidewalls to maintain the rod-shaped cell wall in *M. smegmatis*.

The differential spatial organization of PBPs and LDTs in *M. smegmatis* suggests that the PG composition and its PG-lattice structure may not be uniform throughout the cell but may spatially vary. We envision that the cell walls at the growing poles of *M. smegmatis* primarily consist of 4→3 crosslinked PG-dimer lattice, which is supported by the low abundance of 4→3 crosslinked PG trimers (Fig. 3d). As the cell grows, the PG composition and structure of 4→3 crosslinked cell walls are gradually modified by d,d-endopeptidases and LDTs, transitioning to 3→3 crosslinked PG-trimer lattice structures that are found in the lateral sidewall^31^. For 4→3 crosslinked PG dimers, the ratio of PG disaccharide with an *N*-deacetylation to unmodified to *O*-acetylation is 7:32:9 with MurNGlyc of dimers observed at 26.4% (Table S7). The calculated maximum crosslinking efficiency for a PG-lattice structure that consists entirely of PG dimers is 50%. In comparison, the ratio of 3→3 crosslinked PG trimers with an *N*-deacetylation to unmodified to *O*-acetylation is 10:36:2 with MurNGlyc of trimers measured at 8.9% (Table S7). The calculated maximum crosslinking efficiency for the PG-lattice structure consisting solely of trimers is 67%. Another notable difference between the 4→3 crosslinked dimers and the 3→3 crosslinked trimers is that 3→3 crosslinked trimers show an increase in *N*-deacetylation while a decrease in both *O*-acetylation and *N*-glycolylation. Since *O*-acetylation and *N*-deacetylation modifications of PG disaccharide occur after the nascent PG biosynthesis by PBPs^6,53^, these modifications may be involved in the regulation of cell wall maturation in *M. smegmatis*.

## Methods

### Mycobacterial growth and cell wall isolation

A preculture of *M. smegmatis* (mc^2^155) grown in Middlebrook 7H9 broth (supplemented with 10% albumin, dextrose, and NaCl), 0.05% Tween 80, and 0.5% glycerol, at 37 °C, 150 rpm orbital shaking was used to inoculate flasks containing 500 ml of supplemented 7H9 broth (1% [vol/vol]). Following inoculation, the growth was monitored using a spectrophotometer by measuring optical density at 600 nm at 3 h intervals. Bacterial cells were harvested at a stationary phase (OD_600_, 1.6), approximately 24 h of growth, by centrifugation at 4,750 rpm (Allegra X-15R with SX4750 rotor; Beckman Coulter) for 10 min. The pellets were resuspended in phosphate-buffered saline (PBS) and then immersed in a water bath for 30 min. Cells were agitated and lysed with 0.5-mm-diameter glass beads using Bead Mill 24 (Fisher Scientific) in an alternating cycle of eight 1-min bead-beating with 1-min intervals between agitations for a total of 15 min. Beads and other contaminants from the crude cell wall extract were filtered out using a Steriflip 20-μm nylon vacuum filter (EMD Millipore). Following resuspension in PBS, 2% sodium dodecyl sulfate (SDS) solution was added to 1.5 ml of crude cell wall pellets to the final volume of 10 ml and boiled for 30 min. Boiled cell wall pellets were divided into microcentrifuge tubes and washed five times with deionized water by centrifugation at 10,000 rpm (Allegra X-15R with SX4750 rotor; Beckman Coulter) for 3 min to remove SDS. The resulting isolated cell walls were suspended in 2 ml of 50 mM Tris (pH 8.0) buffer.

### Enzymatic digestion of mycobacterial cell walls for LC–MS

To the crude cell wall suspension, 200 μg of DNase was added and the mixture was incubated at 37 °C for 24 h with 150 rpm orbital shaking, followed by the addition of pronase E (200 μg) for an additional 24 h of incubation at 37 °C and 150 rpm. The digest was placed in a water bath at 80 °C for 30 min to inactivate the pronase E. The cell walls were briefly centrifuged and resuspended in 1 ml of 20 mM Tris buffer. To hydrolyze β-1,4 glycosidic bonds in the PG, isolated cell walls were first digested with 0.5 KU of mutanolysin (Sigma-Aldrich) at room temperature for 24 h followed by an additional 0.5 KU of mutanolysin and incubated for 24 h. The digested cell walls were frozen and lyophilized (Labconco). The lyophilized enzymatically digested samples were then dissolved in 1 ml of 0.375 M sodium borate buffer (pH 9.0) using high-performance liquid chromatography (HPLC)-grade water. Sodium borohydride (10 mg/ml) was added to this preparation and incubated at room temperature for 30 min and quenched by the addition of 125 μl 85% phosphoric acid. The reduced samples were frozen at − 80 °C and then lyophilized. Digested cell wall fragments were diluted in phosphate buffer, and centrifuge filtered through 30 kDa filters at 14,800 rpm for 7 min, followed by second filtration using 0.45 μm filters using similar physical conditions. The isolated cell wall extracts were then lyophilized. Prior to LC–MS analysis, lyophilized samples were resuspended in 1 ml of sample preparation buffer (1% trifluoroacetic acid) and purified for LC–MS using 100-μl Pierce C_18_ tips (Thermo Scientific). C18 tip was prepared by aspirating with a 100µL of wetting buffer (50:50 acetonitrile (ACN): HPLC graded water) followed by a 100µL of equilibrium buffer (0.1% Trifluoroacetic acid (TFA) in HPLC graded water). The analytes were loaded to the C18 tip by slowly aspirating and dispensing 100µL of prepared PG sample for 10 cycles. The tip was rinsed with 100µL of the rinse buffer (0.1% TFA in 5% ACN:HPLC graded water). Lastly, 100µL of elution buffer was aspirated and the purified sample was dispensed into the sample vial.

### High-resolution liquid chromatography-mass spectrometry

Mutanolysin-digested muropeptide fragments of *M. smegmatis* were chromatographically separated using the Waters C18 nano ACQUITY Ultra Performance LC system. A reverse-phase BEH C_18_ column (length, 100 mm; diameter, 75 μm) of a 1.7-μm bead with a pore size of 130 Å was used. Eluents were analyzed with a Waters Synapt G2 high-definition mass spectrometer (HDMS) time-of-flight (TOF) mass analyzer, optimized for an m/z range of 100 to 2,000, operating under positive-ion mode. Nanoflow Electrospray ionization (ESI) was performed on the sample with a spray voltage of 35 V and a capillary voltage of 3.5 kV. Chromatographic separation of mutanolysin-digested PG was performed by injecting 2 μl of the sample from a 5-μl sample loop to the column under isocratic conditions of 98% mobile phase A (0.1% formic acid in HPLC water) and 2% mobile phase B (90% acetonitrile and 10% of HPLC water added with 0.1% formic acid) for 5 min, and then a linear gradient to 50% buffer B was applied for 30 min. The column was regenerated under isocratic conditions with 85% buffer B for 5 min, a linear gradient to 98% buffer A for 1 min, and then isocratic conditions at 98% buffer A for 23 min. The flow rate was kept constant (0.45 μl/min) throughout the analysis. Fibrinopeptide B (Glu-Fib) was used as an internal standard to correct for the drift of the instrument. All measurements were carried out in triplicates using mutanolysin-digested isolated cell walls prepared from a single 500-ml growth culture. Thus, the calculated error bar for a 95% confidence interval represents technical error.

### LC–MS data analysis

Data analysis was carried out using MassLynx (Waters). MATLAB (MathWorks) was used for the calculation of exact masses and isotopic distributions.

## Supplementary Information

Supplementary Information.
