# Supplementary material for: Peptidoglycan compositional analysis of Mycobacterium smegmatis using high-resolution LC–MS
Source: Sci Rep. 2022 Jun 30;12:11061. doi: 10.1038/s41598-022-15324-1 (PMC9247062; doi:10.1038/s41598-022-15324-1)

SUPPORTING INFORMATION

Peptidoglycan compositional analysis of *Mycobacterium smegmatis* using high-resolution LC-MS

Binayak Rimal[1,2], Sibusiso Senzani[3], Christopher Ealand[3], Gyanu Lamichhane[2], Bavesh Kana[3], and Sung Joon Kim[4]**

[1]Institute of Biomedical Studies, Baylor University, Waco, Texas 76798, USA; [2]Johns Hopkins University, School of Medicine, Division of Infectious Diseases, Baltimore, MD 21287, USA; [3]DST/NRF Centre of Excellence for Biomedical TB Research, Faculty of Health Sciences, University of the Witwatersrand, National Health Laboratory Service, Johannesburg, 2001, South Africa; [4]Howard University, Department of Chemistry, Washington, D.C. 20059, USA.

***For Correspondence**: Sung Kim (sung.kim@howard.edu) Tel: 202-806-6900, Department of Chemistry, Chemistry Building, 525 College Street, Howard University, Washington, D.C. 20059, USA; and Bavesh Kana (bavesh.kana@wits.ac.za) University of the Witwatersrand, National Health Laboratory Service, Faculty of Health Sciences, School of Pathology, Johannesburg, 2001, South Africa.

Table S1. Observed fractions of peptidoglycan oligomers found in the cell walls of *M. smegmatis* (mc^{2}155).

PG fragments	*Average (%)*[a]	*Standard deviation*	*CI*[b]
Monomers	16.0266	0.1414	0.3512
Dimers	65.2535	1.3706	3.4051
Trimers	18.7199	1.5049	3.7386

[a]An average determined from triplicate measurements and shown in percentile PG-repeat unit. [b] 95% confidence interval.

Table S2. D-Alanylation and PG crosslinking in mutanolysin-digested cell walls of *M. smegmatis*.

a. PG Fragment	b. Number of D-Alanine	c. Terminal Peptide stem			d. Cross-link (CL)
		Tri	Tetra	Penta	
Monomer	0	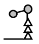			NA
	1		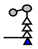		NA
	2			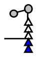	NA
Dimer	0	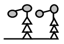			3-3 CL
	1	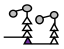	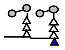		4-3 or 3-3 CL
	2		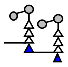	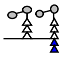	4-3 or 3-3 CL
	3			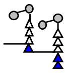	4-3 CL
Trimer	0	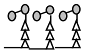			3-3 CL
	1	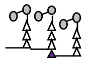<br>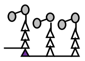	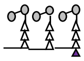		4-3 or 3-3 CL
	2	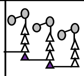	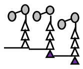<br>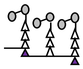	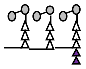	4-3 or 3-3 CL
	3		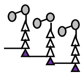	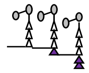<br>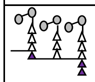	4-3 or 3-3 CL
	4			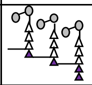	4-3 CL

The column *"a"* categorizes the muropeptides based on the degrees of oligomerization. Column *"b"* is the total number of D-alanine attached to terminal peptide stem in ascending order with the schematic representations of the chemical structure of the corresponding PG fragments with the possible crosslinking are shown in column *"c"*. The total number of D-alanine (blue triangle) is used to identify the types of PG crosslinks, 3-3 or 4-3, as shown in *column d*. In 3-3 crosslinks, a peptide bond formed between the *m*-DAP side chain of a donor stem to the carbonyl carbon on *m*-DAP of an acceptor tripeptide stem. In the 4-3 crosslink, a peptide bond is formed between the *m*-DAP to the D-Ala of the neighboring tetrapeptide stem structure.

Table S3. **D-Alanylation of *M. smegmatis* peptidoglycan as a function of oligomerization.**

	Number of D-Ala	***Average (%)[a]***	***Standard deviation***	***CI [b]***
Monomers	0	10.5746	0.1862	0.4625
	1	61.8070	0.1926	0.4784
	2	27.6184	0.3588	0.8914
Dimers	0	13.9538	0.1933	0.4803
	1	24.2868	0.3248	0.8069
	2	54.4065	0.3270	0.8125
	3	7.3529	0.2134	0.5301
Trimers	0	34.2567	2.2917	5.6934
	1	36.0748	1.7721	4.4025
	2	11.3629	1.8876	4.6894
	3	17.5785	1.9361	4.8099
	4	0.7271	0.1259	0.3128

[a]An average is determined from triplicate measurements and shown in percentile PG-repeat unit. Monomers, dimers, and trimers are each normalized to 100%. [b] 95% confidence interval.

Table S4. The measured PG crosslinkage types in the cell walls of *M. smegmatis*.

	Crosslink (CL)	*Average (%)*[a]	*Standard deviation*	*CI*[b]
Dimers	3→3 CL	13.9538	0.1933	0.4803
	Both CL	78.6933	0.3351	0.8325
	4→3 CL	7.3529	0.2134	0.5301
Trimers	3→3 CL	34.2567	2.2917	5.6934
	Both CL	65.0162	2.4153	6.0005
	4→3 CL	0.7271	0.1259	0.3128

[a]An average is determined from triplicate measurements and shown in percentile PG-repeat unit. Dimers and trimers are each normalized to 100%. [b] 95% confidence interval.

Table S5. The relative ratio of *N*-acetylated to *N*-glycolylated muropeptide from the mutanolysin digested cell walls of *M. smegmatis*.

Muropeptide fragments	*Average (%)*[a]	*Standard deviation*	*CI*[b]
N-acetylated Muramic acid (0)	77.7813	0.1808	0.4491
N-Glycolylated Muramic acid (+1)	22.2187	0.1808	0.4491

[a]An average is determined from triplicate measurements and shown in percentile PG-repeat unit. [b] 95% confidence interval.

Table S6. Calculated average fragment size of unmodified and *N*-glycolylated PG in cell walls of *M. smegmatis*.

	Average fragment size (PG-repeat units)[a]	*Standard deviation*	*CI*[b]
MurNAc (0)	1.7511	0.0165	0.0410
MurNGlyc (+1)	1.9257	0.0046	0.0113

[a]An average is determined from triplicate measurements. [b] 95% confidence interval.

Table S7. PG composition of the observed muropeptide ions from the cell walls of *M. smegmatis* plotted against PG oligomerization.

	Glycolylation of MurNAc	*Average (%)*[a]	*Standard deviation*	*CI*[b]
Monomer	0	12.7163	0.1699	0.4221
	+1	3.3103	0.0808	0.2008
Dimer	0	48.0047	1.2401	3.0808
	+1	17.2488	0.1572	0.3906
Trimer	0	17.0602	1.4666	3.6435
	+1	1.6597	0.0442	0.1099

[a]An average is determined from triplicate measurements and shown in percentile PG-repeat unit. [b] 95% confidence interval.

Table S8. Acetylation of peptidoglycan disaccharide in the cell walls of *M. smegmatis*.

PG acetylation	*Average (%)*[a]	*Standard deviation*	*CI*[b]
N-deacetylated (-1)	15.6736	0.1892	0.4701
Unmodified (0)	56.7246	0.1962	0.4874
O-acetylated (+1)	27.6018	0.0997	0.2478

[a]An average is determined from triplicate measurements and shown in percentile PG-repeat unit. [b] 95% confidence interval.

Table S9. PG composition of trimers based on the acetylation and alanylation states in the cell walls of *M. smegmatis*. (PG-repeat unit)

CL	*Ala*	*Net Acetylation*	*Average (%)*[a]	*Standard deviation*	*CI*[b]
3→3 CL	0	-1	7.0762	0.5556	1.3804
	0	0	25.8609	1.6931	4.2063
	0	1	1.3197	0.0815	0.2026
Both 3→3 and/or 4→3 CL	1	-1	6.7206	1.0377	2.5779
	1	0	29.1203	0.7257	1.8028
	1	1	0.2338	0.0164	0.0408
	2	-1	3.2202	0.5951	1.4783
	2	0	8.1426	1.2967	3.2214
	2	1	0.0000	0.0000	0.0000
	3	-1	2.4658	0.1044	0.2594
	3	0	11.7190	1.2907	3.2066
	3	1	3.3936	0.5489	1.3637
4→3 CL	4	-1	0.2424	0.0420	0.1043
	4	0	0.4847	0.0839	0.2085
	4	1	0.0000	0.0000	0.0000

[a]An average is determined from triplicate measurements and shown in percentile PG-repeat unit. [b] 95% confidence interval.

Table S10. Muropeptide composition of 3→3 CL trimers with varying acetylation states.

Trimers	*Net Acetylation*	*Muropeptide (%)*[a]
3→3 CL	-1	20.66
	0	75.49
	1	3.85

[a]An average is determined from triplicate measurements.

Table S11. Muropeptide composition of 4→3 CL trimers with varying acetylation states.

Trimers	*Net Acetylation*	*Muropeptide (%)*[a]
4→3 CL	-1	33.34
	0	66.66
	1	0.00

[a]An average is determined from triplicate measurements.

Table S12. PG composition of dimers based on the acetylation and alanylation states in the cell walls of *M. smegmatis*. (PG-repeat unit)

CL	*Ala*	*Net Acetylation*	*Average (%)*[a]	*Standard deviation*	*CI*[b]
3→3 CL	0	-1	1.4708	0.0927	0.2302
	0	0	9.4914	0.1087	0.2700
	0	1	2.9916	0.0390	0.0969
Both 3→3 and/or 4→3 CL	1	-1	4.9649	0.1207	0.2998
	1	0	15.3920	0.2595	0.6448
	1	1	3.9299	0.0884	0.2195
	2	-1	6.1590	0.4277	1.0626
	2	0	33.3763	0.1213	0.3013
	2	1	14.8712	0.3524	0.8754
4→3 CL	3	-1	1.0567	0.0767	0.1906
	3	0	4.9588	0.1222	0.3036
	3	1	1.3374	0.1608	0.3994

[a]An average is determined from triplicate measurements and shown in percentile PG-repeat unit. [b] 95% confidence interval.

Table S13. Muropeptide composition of 3→3 CL dimers with varying acetylation states.

Dimers	*Net Acetylation*	*Muropeptide (%)*[a]
3→3 CL	-1	10.54
	0	68.02
	1	21.44

[a]An average is determined from triplicate measurements.

Table S14. Muropeptide composition of 4→3 CL dimers with varying acetylation states.

Dimers	*Net Acetylation*	*Muropeptide (%)*[a]
4→3 CL	-1	14.37
	0	67.44
	1	18.19

[a]An average is determined from triplicate measurements.

Table S15: The calculated and observed masses for the representative muropeptides in Figures 2-5.

Figure	Exact mass (m)		Charge (z)	m/z	
	Calculated	Observed		Calculated	Observed
2a	829.3906	829.3944	2	414.6953	414.6972
2b	1811.8005	1811.7912	3	603.9335	603.9304
2c	2719.2027	2719.2147	3	906.4009	906.4049
3	1699.7368	1699.7376	3	566.5789	566.5792
3	1823.8481	1823.8560	3	607.9494	607.9520
3	1916.8463	1916.8406	2	958.4231	958.4203
4a	895.4124	895.4088	2	447.7062	447.7044
4b	1808.8484	1808.8566	3	602.9495	602.9522
4c	2743.2139	2743.2003	3	914.4046	914.4001
5a	1766.8146	1766.8232	2	883.4073	883.4116
5b	1788.7989	1788.7942	2	894.3995	894.3971
5c	1907.8692	1907.8698	3	635.9564	635.9566

Figure S1. Chemical structures of the representative PG fragments corresponding to the MS spectra shown in Figure 2. The chemical structures of PG dimer and trimer represent one of many possible isomers with a varying position of alanylation.

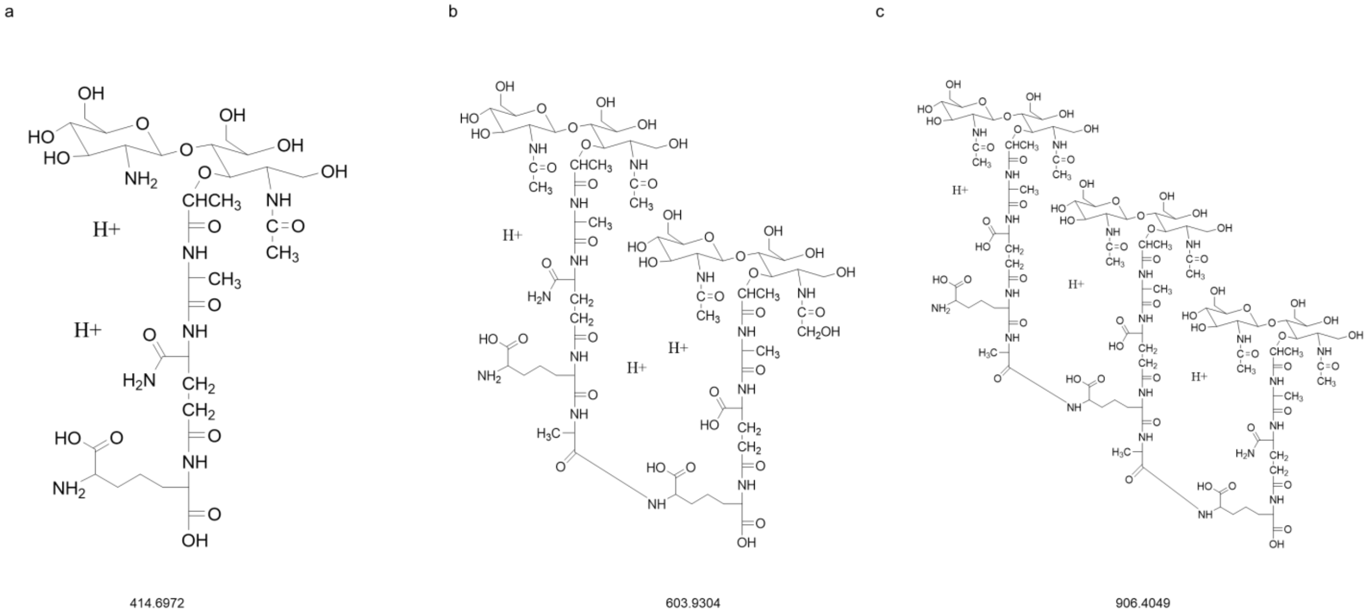

Figure S2. Chemical structures of the representative PG fragments corresponding to the MS spectra shown in Figure 3. The chemical structures of PG dimers represent one of several possible isomers that have varying positions of alanylation as illustrated in Figure 3 inset.

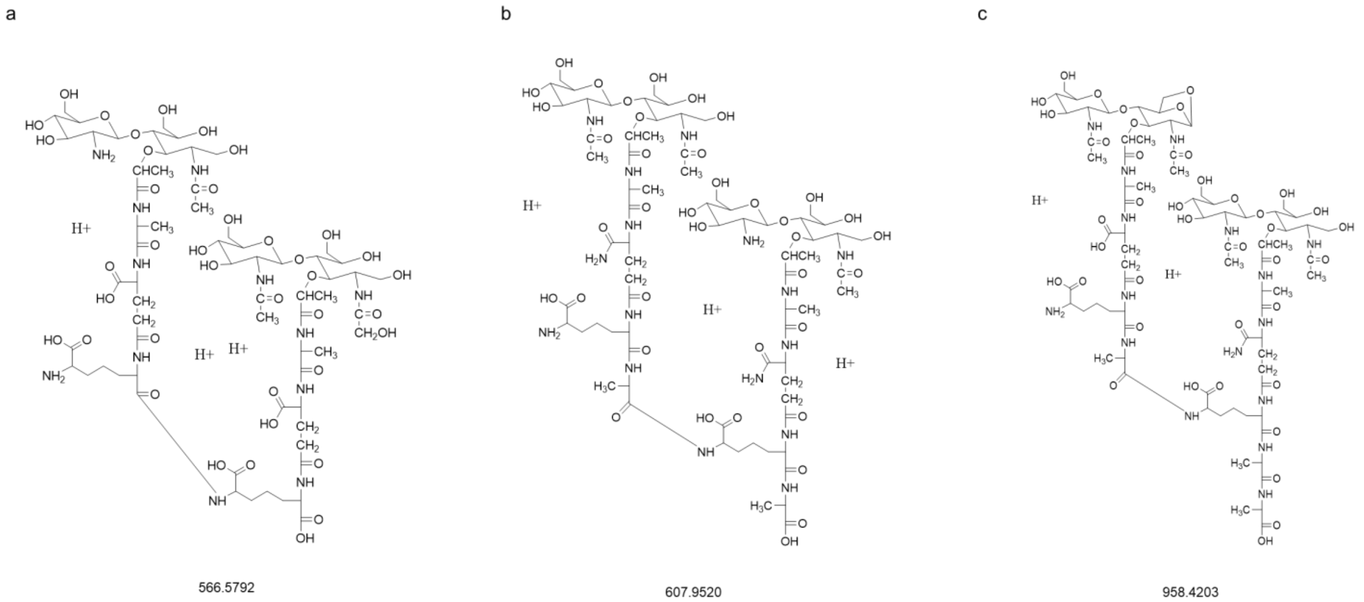

Figure S3. Chemical structures of the representative PG fragments corresponding to the MS spectra shown in Figure 4. The chemical structures of dimer and trimer represent one of many possible structures with a varying position of *N*-glycolylation and anhydro ring structure.

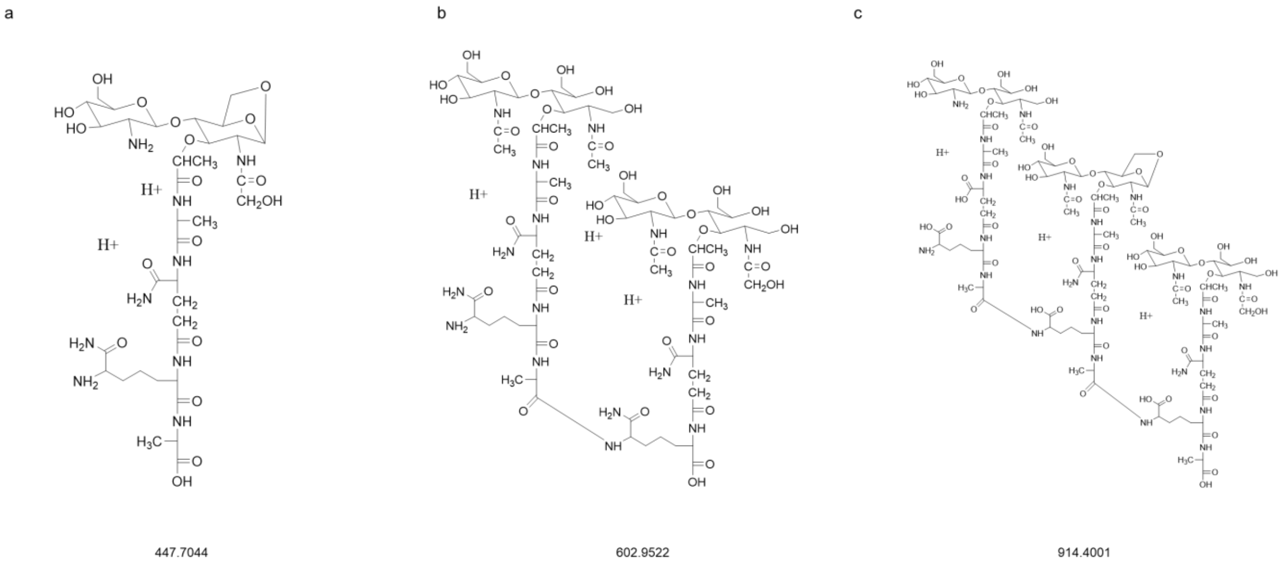

Figure S4. Chemical structures of the representative PG fragments corresponding to the MS spectra shown in Figure 5. The chemical structures of PG dimers represent one of many possible isomers with varying positions of acetylation, deacetylation, and alanylations.

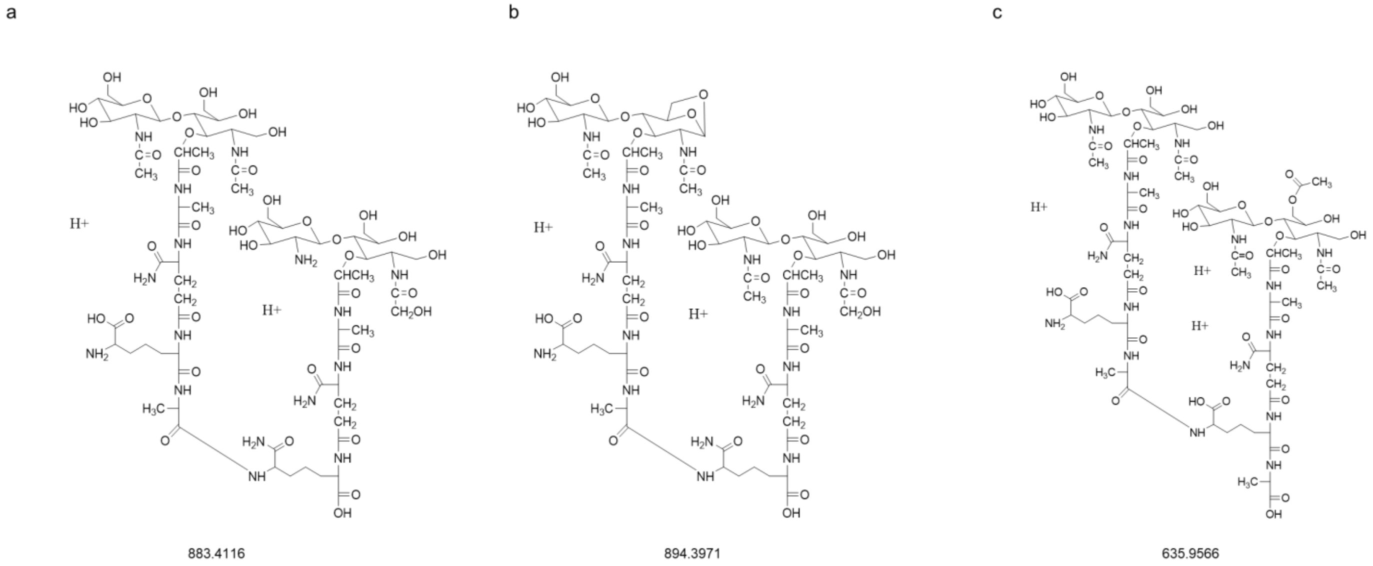

Supplement: Supplementary file 1 — Supplementary Information. [file 41598_2022_15324_MOESM1_ESM.pdf]
